# Effect of Zinc Source and Exogenous Enzymes Supplementation on Zinc Status in Dogs Fed High Phytate Diets

**DOI:** 10.3390/ani10030400

**Published:** 2020-02-29

**Authors:** Ana Margarida Pereira, Margarida Guedes, Elisabete Matos, Edgar Pinto, Agostinho A. Almeida, Marcela A. Segundo, Alexandra Correia, Manuel Vilanova, António J. M. Fonseca, Ana Rita J. Cabrita

**Affiliations:** 1 LAQV, REQUIMTE, Instituto de Ciências Biomédicas de Abel Salazar, Universidade do Porto, R. Jorge Viterbo Ferreira n° 228, 4050-313 Porto, Portugal; anamargaridabp@hotmail.com (A.M.P.); margarida.guedes.m@gmail.com (M.G.); ajfonseca@icbas.up.pt (A.J.M.F.); 2 SORGAL, Sociedade de Óleos e Rações S.A., Estrada Nacional 109 Lugar da Pardala, 3880-728 S. João Ovar, Portugal; elisabete.matos@sojadeportugal.pt; 3 LAQV, REQUIMTE, Departamento de Ciências Químicas, Faculdade de Farmácia, Universidade do Porto, R. Jorge Viterbo Ferreira n° 228, 4050-313 Porto, Portugal; edgarpinto7@gmail.com (E.P.); aalmeida@ff.up.pt (A.A.A.); msegundo@ff.up.pt (M.A.S.); 4 i3S/IBMC—Instituto de Investigação e Inovação em Saúde/Instituto de Biologia Molecular e Celular, Universidade do Porto, R. Alfredo Allen, 4200-135 Porto, Portugal; alexandra.correia@ibmc.up.pt (A.C.); vilanova@icbas.up.pt (M.V.); 5 Instituto de Ciências Biomédicas de Abel Salazar, Universidade do Porto, R. Jorge Viterbo Ferreira n° 228, 4050-313 Porto, Portugal

**Keywords:** zinc sulfate, zinc proteinate, dogs, solid-state fermentation product of *Aspergillus niger*, phytates

## Abstract

**Simple Summary:**

Pet food is a sector in expansion, demanding novel solutions for old issues. The mineral supplementation of animal feed has been a target of research for livestock animals, but less attention has been devoted to companion animals. The present study offers both nutritionists and pet food manufacturers knowledge concerning organic and inorganic sources of zinc for dog food supplementation. Zinc proteinate and zinc sulfate were added to a high phytate dry food for healthy dogs at maintenance. Moreover, the role of exogenous enzymes to overcome the undesirable effects of phytates was also studied, with the inclusion of a solid-state fermentation product from *Aspergillus niger*. Data suggest that organic zinc is promising for dog food supplementation. Yet, the use of exogenous enzymes seems to require refinement to fit the needs and physiological conditions of dogs and thus add value to mineral availability and nutrient digestibility.

**Abstract:**

Zinc is an essential element, a cofactor of many enzymes, and performs catalytic, structural and regulatory functions. Once in the gastrointestinal tract, zinc can interact with food constituents. Phytic acid, the major phosphorus storage in plants, limits zinc availability from animal feeds due to the formation of insoluble complexes with phytates. This study tested the effect of supplemental zinc source (zinc sulfate and a chelate zinc proteinate) and the addition of exogenous enzymes from a solid-state fermentation product of *Aspergillus niger* to a high phytate diet. The study was designed according to three Latin Squares 4 × 4 with a 2 × 2 factorial arrangement of treatments, with four periods, four diets, and 12 young adult Beagles. Periods lasted 5 weeks each. Diets were supplemented with 75 mg/kg of zinc sulfate (IZ) or zinc proteinate (OZ), and without or with 200 mg/kg of exogenous enzymes (IZ+, OZ+). Results showed that zinc proteinate increased the bioavailability of phosphorus, yet the zinc biomarkers remained unaffected by the zinc source, with the exception of lymphocyte subsets that benefit from zinc proteinate. The use of exogenous enzymes did not affect zinc availability nor nutrient and energy digestibility.

## 1. Introduction

Zinc is a recognized essential element for all organisms, a cofactor of several enzymes, and performs catalytic, structural and regulatory functions [1]. In dogs, it is well known that Zn deficiency, among others, decreases Zn serum concentration, the activity of plasma and tissue Zn-requiring enzymes and growth rate. Moreover, it causes skin lesions, behavioral problems, thymus atrophy and compromises immune function [2,3]. Zinc deficiency might be a consequence of low dietary intake, genetically-based Zn malabsorption or the interference of dietary constituents with the absorption of Zn [3]. The National Research Council (NRC) recommends a minimum dietary allowance of Zn of 15 mg per 1000 kcal of metabolizable energy (ME) for adult dogs in maintenance, 11 mg per 1000 kcal of ME for puppies, and 24 mg per 1000 kcal of ME for bitches in late gestation and peak lactation [1]. Even with dietary Zn levels above this recommended minimum, symptoms of Zn deficiency may occur, as the availability of Zn is not merely dependent on its own dietary content [4], but also on its interaction with other elements, especially Cu, Fe, and Ca, and the presence of dietary antagonists.

Phytic acid (PA) is considered the major dietary constituent that limits Zn availability due to the formation of insoluble complexes [5]. Phytic acid is the main form of P storage in plants, and is widely found in cereals, legume seeds, oilseeds, and cereal by-products. 

Dietary fibers might also decrease Zn bioavailability due to the formation of insoluble chelates [6]. Dietary fiber is traditionally used in dog foods to promote stool quality and is comprised of the plant components of the diet that are not digested by endogenous enzymes but are fermented in the large intestine with the production of gases and volatile fatty acids. Non-starch polysaccharides (NSP) are a component of dietary fiber and comprise soluble and insoluble fractions. Soluble NSP become viscous in solution, inhibiting endogenous enzymes to interact with nutrients, compromising nutrient digestibility [6]. Despite being important for bacterial biomass, an excessive fermentation of soluble NSP in the large intestine induces poor fecal quality [7]. Insoluble NSP are important to bulk feces, but can also reduce nutrient and mineral digestibility by setting nutrients apart from endogenous enzymes and by binding minerals within polysaccharide and phytate molecules [6]. The addition of exogenous enzymes has been used as a strategy to reduce the undesired effects of fiber and phytate and thus improve feed efficiency in fish and broillers [8,9].

The magnitude of the effect of dietary antagonists and interaction with other elements on Zn bioavailability may be influenced by the source of supplemental Zn. It is well known that inorganic sources of Zn, such as Zn oxide or Zn sulfate, commonly used in dog food supplementation, are more susceptible to interaction with other dietary constituents when compared to organic forms of Zn [10], whose bioavailability may depend on the nature of the organic form [11]. The greater absorption of organic Zn sources as Zn amino-acid chelate [12] and Zn propionate [4], reported in dogs, is more likely to ensure the Zn requirements of animals with a lower dietary Zn supplementation. Since the level of inclusion of Zn in the diet is legally limited, this feature of organic sources is particularly important in conditions of high Zn needs such as growth, gestation or illness [13].

The present study aimed to evaluate, for the first time, the effects of Zn source (Zn proteinate and Zn sulfate) and the addition of a solid-state fermentation product of *Aspergillus niger* with residual enzymatic activity on the Zn status of dogs fed diets with a high phytate content. 

## 2. Materials and Methods

### 2.1. Animals and Management

The trial was approved by the Animal Ethics Committee of Abel Salazar Institute of Biomedical Sciences, University of Porto (ICBAS-UP) and licensed by the Portuguese Directorate-General of Food and Veterinary Medicine (permit N. ° 206/2017). All the procedures were carried out by trained scientists in research animal care (FELASA category C) that conducted the experiments in line with the guidelines from the abovementioned institutions.

Twelve one-year-old Beagle dogs, six males and six females, (11.3 ± 1.1 kg of body weight, BW) participated in the trial. The sample size was calculated using power analysis, assuming a power of 95% and a *p*-value of 0.05 from data previously published by Wedekind and Lowry (1998) [4], and Tortola et al. [14], which evaluated Zn sources for dog food supplementation and addition of exogenous enzymes to enhance nutrient digestibility, respectively. All dogs were subjected to a clinical examination to check the suitability to participate in the trial. At night, dogs were housed in pairs in communicating boxes with interior and exterior areas of 1.8 and 3.5 m^2^, respectively. During the day, dogs had access to an 85 m^2^ outdoor park to exercise and socialize. Leash walks were daily assured as part of environmental enrichment. The temperature and relative humidity of the kennel were monitored daily. 

Dogs were individually fed their daily ration in two meals (9 am and 5 pm) and freshwater was provided ad libitum. The daily food amount was calculated to meet the metabolizable energy (ME) requirements according to dogs’ BW, using the following equation $\mathrm{ME}\;\left(\mathrm{Mj}\right)=0.544\times BW^{0.75}$ [1], and adjusted for body condition score (BCS; 9-point scale) [15]. Both BW and BCS were recorded weekly. When present, the leftovers were weighed to calculate daily food consumption.

### 2.2. Diets and Experimental Design

The trial was designed according to three Latin Squares 4 × 4 with a 2 × 2 factorial arrangement of treatments, with four periods and four diets, in which all dogs were subjected to all treatments. Each period comprised four weeks for diet adaptation and one week for data collection. Four complete dry dog foods were formulated to meet the nutrient and ME requirements of adult dogs only differing in the source of supplemental Zn and with or without the addition of exogenous enzymes (Table 1): (1) inorganic Zn in the form of Zn sulfate monohydrate, and no enzyme addition (diet IZ); (2) inorganic Zn in the form of Zn sulfate monohydrate with enzyme addition (diet IZ+); (3) organic Zn in the form of a chelate Zn proteinate (Bioplex^®^ Zinc, Alltech, KY. USA) and no enzyme addition (diet OZ); and (4) organic Zn in the form of a chelate Zn proteinate (Bioplex^®^ Zinc) with enzyme addition (diet OZ+). The content of Zn and PA in the individual ingredients was previously analyzed (Table 1) to formulate a diet with a high phytate content (> 9 mg/kg) [16], and a level of Zn that meets requirements respecting the legal impositions. Thus, Zn supplementation of all the four diets was 75 mg/kg regardless of the form used, which corresponds to ≈ 40% of the total dietary content of Zn. A commercial multi-enzymatic complex (including phytase, protease, xylanase, ß-glucanase, cellulase, amylase and pectinase) from the solid-state fermentation of *A niger* (Synergen^®^, Alltech Inc, Nicholasville, KY, USA) was added in the coating to the IZ+ and OZ+ diets at a level of 200 mg/kg.

### 2.3. Sample Collection and Processing

Blood and urine samples from each dog were collected on the third day of weeks 5, 10, 15 and 20 of the trial. Urine (pre-meal) was collected directly into sterilized containers by voluntary urination for urinalysis. Additionally, two spot samples of urine from the same day were collected and pooled in sterilized plastic containers for the determination of creatinine (CT) and stored at −20 °C for further trace elements analysis.

Blood was collected by venipuncture before the morning meal into VACUETTE^®^ EDTA (Greiner Bio-one, Kremsmunster, Austria), VACUETTE^®^ Clot Activator gel (Greiner Bio-one, Kremsmunster, Austria), VACUETTE^®^ Lithium Heparin (Greiner Bio-one, Kremsmunster, Austria), and BD Vacutainer^®^ Trace Element and Lead tubes (Becton Dickinson, Vaud. Switzerland) for hemogram, serum biochemistry, white blood cell (WBC) isolation and trace elemental analysis, respectively. Serum chemistry was performed on the same day, and aliquots were stored at −20 °C and −80 °C for further serum C-reactive protein (CRP) and serum superoxide dismutase (SOD) activity analysis, respectively.

An area of 5 × 5 cm located at the lateral left cranial region was shaved before the beginning of the trial and at weeks 5, 10, 15 and 20 to allow hair collection for Zn determination. Hairs were washed using a procedure adapted from Morton et al. [17]. The hairs were placed in 50 mL-conical tubes, 3 mL of 14 mM Triton X-100 (Sigma-Aldrich, St. Louis, MO, USA ) was added and tubes were left at a shaken platform for 3 h. The solution was pipetted off and 4.5 mL of ultrapure water was added and shaken for another 2 h. Hairs were then rinsed three times with ultrapure water and allowed to dry in a forced-air oven at 65 °C for 48 h inside the conical tubes without the cap.

### 2.4. Analytical Methods

#### 2.4.1. Urinalysis, Hemogram, and Serum Chemistry

Urine color and turbidity were evaluated as recommended by WSAVA [18]. Urinalysis was obtained with a dipstick, pH and density were determined with a potentiometer (pH and Ion-Meter GLP 22, Crison, Barcelona, Spain) and a refractometer, respectively. Hemogram was performed using a hematology analyzer (Sysmex XT-2000iV, Norderstedt, Germany). The serum chemistry profile and urinary CT were determined using a chemistry analyzer (Mindray BS-380, Shenzhen, China). Serum CRP was measured by immunoturbidimetry using a Roche Cobas c501 analyzer (Roche Diagnostics, Basel, Switzerland) with the Gentian Canine CRP Kit (Gentian Diagnostics, Stockholm, Sweden). Serum SOD activity was determined using the SOD Assay Kit (Enzo, New York, NY, USA) following the manufacturer’s instructions and a Cytation 3 microplate reader (Bio-Tek Instruments, Winooski, VT, USA) controlled by Gen 5 software (Bio-Tek Instruments, Winooski, VT, USA) for colorimetric detection. The value of Cu/ZnSOD activity was expressed as units per g of serum total protein (TP). 

#### 2.4.2. Diets

Diet samples were dried for 48 h at 65 °C in an air-forced oven and further ground (1 mm) for the determination of dry matter (DM) [19], ash [20], ether extract (EE) [20], Kjeldahl N [21], neutral detergent fiber (NDF, assayed with α-amylase and expressed of exclusive ash), acid detergent fiber (ADF) and acid detergent lignin (ADL) [22]. Crude protein (CP) was calculated as Kjeldahl N × 6.25. The starch content was determined on 0.5 mm- ground samples [23]. The gross energy (GE) was determined using an adiabatic bomb calorimeter (Werke C2000, IKA, Staufen, Germany). Total P and PA were analyzed using the Phytic Acid Assay Kit (Megazyme, Bray, Ireland), after overnight incubation in a shaken platform of 1 g of food/ingredient in 0.6 M HCl at room temperature. The manufacturer’s protocol was followed, and the colorimetric detection was performed using a Cytation 3 microplate reader controlled by Gen 5 software. For analytical control, certified reference material, oat flour powder supplied with the commercial kit, was assayed along with the samples. The results are presented as Supplementary Material in Table S1. 

#### 2.4.3. Elemental Analysis

High-purity reagents from Sigma-Aldrich (St. Louis, MO, USA) and ultrapure water (18.2 MΩ cm), from an Arium^®^ water purification system (Sartorius, Goettingen, Germany) were used in all analysis. Plasticware was immersed overnight in 10% HNO_3_ for decontamination and rinsed with ultrapure water before use.

Dried (65 °C, 48 h) and milled (1 mm) food/ingredient samples were solubilized by microwave-assisted acid digestion with 3 mL of HNO_3_ and 1 mL of H_2_O_2_. Na, K, Ca, Mg Zn, and Fe were determined by flame atomic absorption spectroscopy (FAAS) with an AAnalyst 200 flame (air-acetylene) atomic absorption spectrometer (PerkinElmer, Überlingen, Germany). Se, Mn, and Cu, were determined by inductively coupled plasma mass spectrometry (ICP-MS) using an iCAP Q (Thermo Fisher Scientific, Schwerte, Germany) instrument, equipped with a MicroMist nebulizer, a Peltier cooled cyclonic spray chamber, a standard quartz torch, and Ni skimmer, and sampling cones. The methodology of analysis is further detailed by Pereira et al. [24]. 

Plasma and urine samples were diluted to 1:10 with a solution containing 130 mM HNO_3_, 0.17 mM Triton X-100, 54 mM butanol, and Rh (internal standard) as suggested by Goulle et al. [25]. Synthetic urine (92 mM NaCl, 5 mM K, 29 mM Mg, 5 mM Ca, 270 mM Cl, 15 mM SO_4_, and 280 mM CH_4_N_2_O) was added to the calibration standards in the urine analysis. The determination of trace elements in both urine and plasma was performed by ICP-MS under the same conditions previously described for food analysis. Results of trace elements in urine and plasma were expressed as μg/g of CT and as mg/g of CT, respectively. 

For the determination of Zn in hair, 180 ± 60 mg of sample was weighed to PTFE vessels and digested in a microwave digestion unit (Milestone, Sorisole, Italy) with 2 mL of HNO_3_ and 1 mL of H_2_O_2_, under the following program: 5 min at 250 W; 2 min at 0 W; 5 min at 400 W; 2 min at 0 W; and 5 min at 600 W [17]. Ultrapure water was added to the solubilized material to bring the total volume to 10 mL, further diluted to 1:10 with ultrapure water and analyzed by FAAS using a hollow cathode lamp (PerkinElmer) as radiation source.

For analytical quality control, ≈ 500 mg of BCR^®^-679 white cabbage and ≈ 200 mg of ERM^®^-DB001 human hair (Institute for Reference Materials and Measurements, Geel, Belgium) were digested and analyzed following the same procedures as for food and dog hair samples, respectively (Table S1). Additionally, for the analytical control of urine and plasma mineral analysis, Seronorm™ Trace Elements Urine and Seronorm™ Trace Elements Serum (Sero, Billingstad, Norway) were diluted and analyzed as the samples (see Supplementary Material, Table S2).

#### 2.4.4. Flow Cytometry for Determination of CD4/CD8 Ratio

Whole blood was centrifuged (400× *g* for 15 min) and the recovered plasma stored at −20 °C. The erythrocytes of the buffy-coat were lysed with ACK lysis buffer (150 mM NH_4_Cl, 10 mM KHCO_3_, 0.1 mM Na_2_EDTA) to allow the isolation of WBC. Cells were washed twice with Hank’s balanced salt solution (Sigma-Aldrich, St. Louis, MO, USA) and re-suspended in Fluorescence-activated cell sorter (FACS) buffer (phosphate buffer saline, 10 mM of NaN_3_, 10 g/L of bovine serum albumin). For staining, 10^6^ cells were incubated for 30 min with a mix containing the antibody panel (Table 2), washed twice and then re-suspended in FACS buffer. Cells were transferred to cytometry tubes and 0.5 µL of propidium iodide (100 μg/mL)was added to allow the identification of dead cells. Data were collected using a FACSCanto™ II system (BD Biosciences, San Jose, CA, USA) and analyzed with FlowJo™ 10 software (Treestar, Ashland, OR, USA). Beads were used for compensation. The gating strategy used for flow data analysis is shown in Supplementary Material, Figure S1). Fluorescence minus one (FMO) control stainings were done to help defining gates.

### 2.5. Total Tract Apparent Digestibility

Dogs were housed individually for five days at weeks 5, 10, 15 and 20 to allow total feces collection. Individual fecal samples were scored using a 5-point scale (from 1, corresponding to a liquid stool, to 5, corresponding to dry and hard feces [26]), weighed, placed in plastic bags and stored at −20 °C until further analysis. A representative sample from thawed samples was immediately analyzed for Kjeldahl N to allow CP calculation (Kjeldahl N × 6.25). Ash, ether extract, NDF, ADF, starch, GE and P were analyzed from dried (65 °C) and milled (1 mm) feces pooled per dog and period. The coefficient of total tract apparent digestibility (CTTAD; g/kg) was calculated as follows:

(1) $$\frac{\mathrm{nutrient}\;\mathrm{intake}-\mathrm{fecal}\;\mathrm{nutrient}\;\mathrm{output}}{\mathrm{nutrient}\;\mathrm{intake}}.$$

### 2.6. Evaluation of Coat Quality and Trichogram

On the first day of weeks 5, 10, 15 and 20, the evaluation of coat quality was performed by a naïve panel of six elements unaware of the diets (1 animal nutritionist, 2 dog tutors, 1 dog trainer and 2 veterinarians). A day before the coat evaluation, all dogs were washed using a hypoallergenic dog shampoo (VIVOG Universal, Paris, France) to allow a clear visual and sensory judgment by the panel. The panel evaluated the softness, greasiness, and scale of hair as defined by Marsh et al. [27] using a 5-point scale proposed by Hester, in which 1 is the worst and 5 is the best [28]. In order to assess hair growth phases, hairs from three areas (antebrachium, external mid-thigh, and dorsal mid-shoulders) were carefully epilated using forceps. The morphology of about 100 hairs including roots was evaluated through microscopical observation and the percent of hairs in the anagen, catagen or telogen phases was calculated [29]. 

### 2.7. Evaluation of Flatulence

**Figure 1:**
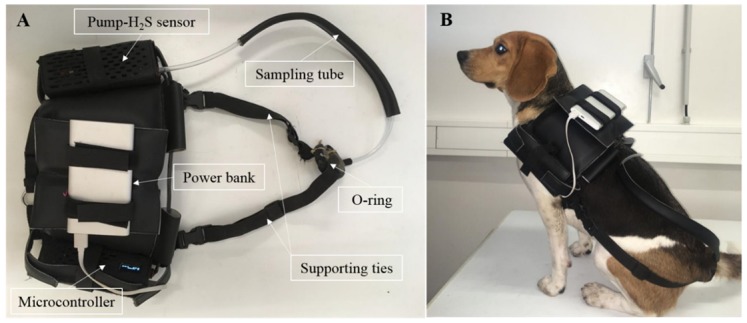
Real image of the vest assembled device used for flatulence assessment based on H_2_S measurement in the anal region (**A**); dog wearing the vest (**B**).

The assessment of dog’s flatulence was performed during 5 h (from 10:30 am until 3:30 pm) in one day at weeks 5, 10, 15 and 20 using a device resembling the one described by Collins et al. [30]. The variables evaluated were the number of flatulence episodes, the maximum and the average intensity of H_2_S detected in each episode. A dog vest was developed to hold the device hardware which consists of an air-pump fitted with an H_2_S sensor connected to a microcontroller both powered by a power bank. Gases were collected from the anal area through a 55 cm-length plastic tube (inner diameter 0.5 cm) connected to the pump-sensor box and an O-ring placed at the base of the tail. An embed harness and abdominal ties supported the vest in position (Figure 1). The weight of the device was 840 g. The H_2_S concentrations were presented in parts per million and recorded every 2 s. The signal was acquired and processed by the microcontroller and stored in an external storage system (SD card). The data was exported as an XLSX file and analyzed offline. Due to the sensitivity of the sensor and the possibility of cross-reactivity with other gases, the assembled vest sensor was allowed to detect and record blank data in the room where the measures took place for 5 h. The blank data was used to distinguish the signal (flatulence episodes) from noise. The threshold for flatulence detection was set for values > 5 ppm registered at least during 10 s. From those, only episodes with a peak of H_2_S ≥ 6 ppm were considered.

### 2.8. Statistical Analysis

Data were subjected to a least-squares ANOVA for three Latin Squares 4 × 4 with a 2 × 2 factorial arrangement of treatments using the general linear model procedure of SAS (SAS^®^ University Edition 2019, Cary, NC, USA). The model included the fixed effects of the square, dog within the square, period, Zn source (Zn), enzyme addition (Enzyme), the interaction of Zn source and enzyme addition (Zn × Enzyme) and the residual error. Whenever the effects were significant, the Tukey test was used to compare means. The statistical level of significance was considered for *p* < 0.05, while the trend was set for *p* < 0.1.

## 3. Results

### 3.1. Diets, Intake and Total Tract Apparent Digestibility

The chemical composition of the experimental diets is presented in Table 3. On average, diets presented 283 g CP/kg DM, 10.3 g PA/kg DM, and 147 mg Zn/kg DM.

Mean BCS was not affected by Zn source nor by enzymes addition, the values ranging from 4.4 to 4.6 (Table 4). The DM intake tended to be affected by Zn source (*p* = 0.063) and was significantly affected by the interaction between Zn source and enzyme addition (*p* = 0.032; Table 4). The addition of exogenous enzymes to the IZ diet decreased daily DM intake, whereas no effect was observed with the OZ diet. Diets supplemented with inorganic Zn promoted an increase in OM (*p* = 0.044), starch (*p* < 0.001), and EE (*p* < 0.001) intake when compared to OZ diets. Enzyme addition decreased the intake of EE (*p* = 0.031) and of P (*p* < 0.001), regardless of the Zn source. The interaction between Zn source and enzyme addition was statistically significant for the intake of NDF, Fe, Cu, Mn, and Zn (*p* < 0.05). Zn intake was lower with diet IZ+ than diet IZ, but was higher in OZ+ than with OZ (*p* < 0.001). Daily intake of CP (*p* = 0.056) and ME (*p* = 0.079) tended to decrease with the addition of enzymes to IZ diet, whereas it tended to increase when enzymes were added to OZ diet.

Zinc source tended to affect DM (*p* = 0.078) and OM digestibilities (*p* = 0.076) with higher values observed for IZ diets (Table 5). Conversely, P digestibility was significantly higher with OZ than IZ diets (*p* = 0.009). Enzyme addition did not affect DM, nutrients and energy digestibility (*p* > 0.05). The interaction between Zn source and enzyme addition affected NDF digestibility (*p* = 0.013), the lowest and highest values being observed with IZ and IZ+ diets, respectively. Fecal score and dried fecal production were similar for all diets (*p* > 0.05; Table 5).

### 3.2. Zinc Biomarkers

Table 6 presents the plasmatic concentration of trace elements and biomarkers of Zn status. Iron was the element with higher plasmatic concentration followed by Zn, Cu, and Mn, but none of them were affected by Zn source nor enzyme addition (*p* > 0.05). Similarly, Zn source and enzyme addition did not influence urinary and hair Zn concentrations (*p* > 0.05). However, the concentration of Zn in hair tended to be higher when enzymes were added (*p* = 0.075). Additionally, Zn source and enzyme addition did not affect SOD activity, serum CRP concentration (*p* > 0.05; Table 6), urine pH and density, hemogram, and serum biochemistry (*p* > 0.05; data not shown), except alanine aminotransferase (ALT), which was significantly higher in dogs fed OZ than IZ diets (*p* = 0.035; Table 6). The percentage of CD4 was affected the Zn source (*p* = 0.041; Table 6), being higher in dogs fed OZ diets.

Results of coat evaluation and trichogram are presented in Table 7. The OZ diets tended to increase the gloss score (*p* = 0.061). On average, scores were 3.8, 4.0, and 3.9, respectively, for softness, greasiness and scale, none of the parameters being affected by Zn source and enzyme addition (*p* > 0.05). Similarly, there were no differences in hair growth (percentage of hairs in anagen, catagen and telogen phases) between diets (*p* > 0.05). However, the interaction between Zn source and enzyme addition tended to affect percentage of hairs in telogen phase (*p* = 0.065).

### 3.3. Flatulence

The flatulence assessment is presented in Table 8. The number of episodes detected in 5 h was highly variable, averaging 3.3 with OZ+, 6.0 for OZ and IZ+ and 10.3 for IZ diet. Nevertheless, the difference in episodes detected was not statistically significant for any experimental diet (*p* > 0.05). The average and maximum intensity of episodes (H_2_S ppm detected) ranged from 22.0 to 17.0 ppm and from 20.4 to 33.8 ppm, respectively. No significant differences were detected for Zn source nor enzyme addition (*p* > 0.05).

## 4. Discussion

### 4.1. Food Composition and Intake

The present study tested the hypothesis that the interference of some food constituents on the bioavailability of Zn supplied at practical levels could be overcome either by the usage of organic supplemental zinc (chelate Zn proteinate, Bioplex^®^) or by the addition of exogenous enzymes from a solid-state fermentation product of *A. niger* (Synergen^®^). The experimental diets simultaneously covered the requirements in macro and microelements for young adults and the legal limits [1]. The dietary content of Zn averaged 147 mg/kg DM, ranging from 139 to 159 mg/kg DM, which is within the European maximum legal limit of 227 mg/kg DM [31]. The amount of supplemental Zn was defined from the Zn content provided by the ingredients and such that the total Zn content was within the range normally found in commercial foods. Indeed, in a total of 162 samples of complete dry dog foods from 22 European countries, the median Zn content was 157.5 mg/kg [32]. Similarly, Kelly et al. reported a median Zn content of 140 mg/kg DM in 18 dry dog foods [33], while Pereira et al. obtained a much higher median, of 310 mg/kg DM, for 20 samples of dry dog food belonging to different market segments [24]. In the present study, the amount of dietary Zn supplied (5.95 to 7.15 mg/kg BW^0.75^; Table 4) was higher than the minimum requirement (2 mg/kg BW^0.75^) established by the NRC for adult dogs in maintenance [1]. Actually, the minimum requirement of dogs would have been ensured by the Zn background level determined in the experimental diets (3 mg/kg BW^0.75^), which is defined as the Zn concentration in the complete food delivered by the food ingredients and agreed with those reported by EFSA obtained from CVB feed tables and by data submitted by the industry [32]. However, the bioavailability of the native Zn in the experimental diets might have been compromised, as the majority of Zn and PA are sourced by wheat and soy concentrate, and as is well known, in cereal and oilseed based diets, the antagonism exerted by phytates applies to native Zn, already bound to it [34]. Additionally, the considerable interactions between Zn, Ca, Cu and Fe and the impact of fibers on Zn availability through the formation of insoluble complexes must be considered for the definition of the optimal dietary amount of Zn. Due to the difficulties in estimating the Zn bioavailability, in practical situations, the background Zn of the basal diet is assumed to be not available [32], being the supplementation with a bioavailable Zn source essential to ensure the requirements. All animals remained healthy throughout the study. The intake of DM tended to be higher in dogs fed IZ. The daily food provided was adjusted to ensure an ideal BCS of the dogs in all groups. The observed effects on nutrients intake reflect the slight differences in DM intake and on the chemical composition of diets. 

### 4.2. Effect of Zn Source

Zinc, as other cations, is prone to interact with food components in the gastrointestinal tract and thus form insoluble complexes that lower its bioavailability. The pH, especially in the proximal part of the digestive tract, influences Zn solubility and availability. Inorganic Zn, such as Zn sulfate, is easily dissociated at the acidic pH of the stomach, therefore Zn^2+^ remains soluble. When it passes to the intestinal compartment, Zn^2+^ can either be bonded to amino acids from the chyme or to carrier proteins of the luminal membranes of the mucosa cells to be transported by passive diffusion or active transport into the bloodstream [35]. However, it can also form insoluble complexes with food constituents such as PA. The solubility of PA is influenced by pH and it is mostly found negatively charged at pH 3–7. Thus, in the intestine, the chelating effect of phosphate groups causes PA to form insoluble complexes with Zn^2+^ and other metal cations [36]. Conversely, as the pH of the stomach does not affect the coordinate covalent bond between Zn and the organic molecule, Zn chelates are not ionized before absorption, and they are transported across the intestinal barrier using the same mechanism as low molecular weight peptides, being the metal ion separated from the organic molecule only at the site of use [35].

The results of the present study showed that the digestibility of P was enhanced with Zn proteinate supplementation. Two studies reported that over-supplementation with Zn-oxide decreased P digestibility in pigs [37,38], suggesting that P uptake was decreased by inorganic Zn, a phenomenon that was already described in plants [38], or that precipitation in the intestinal lumen of P and Zn might have occurred. However, in the present study, this is not supported by the zinc biomarkers evaluated and is unlikely to have occurred with the lower level of Zn supplied compared to the earlier studies. Nevertheless, it seems that Zn from Zn proteinate did not interact with P, which may have positive implications for Zn bioavailability. Additionally, it has been suggested that metal proteinates have lower inhibitory effects on phytases than inorganic trace elements or other organic sources [39] contributing to a higher P digestibility with organic Zn source. In the present study, despite P digestibility have been higher with organic Zn source, no differences were observed between sources when exogenous enzymes were added (interaction between Zn source and enzyme not significant). 

Aside from the interference of anti-nutritional components of the diet, Zn absorption can be impaired by the interaction with other minerals. Nevertheless, mineral interactions tend to be more deleterious if any is supplied in excess [40]. Results described here corroborate this, as no differences in the plasma concentration of Zn and other trace elements were observed following an adequate mineral intake. Urinary Zn was similar among dogs fed Zn sulfate and Zn proteinate, which is not surprising since variations on urinary Zn excretion are associated with its excessive intake/deficiency or with the presence of concomitant diseases [41]. Additionally, urinary excretion is not the preferred route for the control of Zn status, but it is in the gastrointestinal system that the major homeostasis of Zn takes place through the control of absorption of dietary Zn, secretion, and reabsorption of endogenous Zn from pancreatic and biliary and gastroduodenal secretions [42].

Zinc content in hair and overall coat quality and hair growth were similar in dogs fed Zn sulfate and Zn proteinate. Similarly, Kuhlman and Rompala found no significant differences in Zn hair content among dogs fed inorganic and organic sources of Zn, Mn and Cu, but smoother and less fragmented hair follicles on dogs fed the organic sources [43]. However, it is not clear whether these findings are only attributable to Zn source or to the replacement of inorganic sources of all three trace elements by organic ones. In another study from Lowe and Wiseman, dogs were subjected to a 30-day adaptation period with no dietary Zn supplementation (only 56 mg Zn/kg from raw materials) and then fed for another 60 days three levels (50, 75 and 100 mg/kg) of three supplementary sources (Zn proteinate, Zn oxide and Zn polysaccharide), with results showing increased growth rate and Zn deposited in hair for Zn proteinate [43]. This result suggests that Zn proteinate was more efficient in restoring the Zn levels, yet results are not comparable to the ones reported here since the study departs from a Zn depletion status, which may alter the hair retention response to Zn forms. The response of Zn biomarkers to dietary supplementation is not always consistent. When the Zn status is up to a suboptimal level, biomarkers tend to respond positively, enabling to distinguish bioavailability of supplemented forms, as the amount of Zn absorbed and retained increases linearly with supplied Zn. Conversely, when dietary supply exceeds requirements, the amount of Zn absorbed and retained (expressed as a proportion of the ingested Zn amount) decreases with the increase in ingested Zn [44]. 

Along with Zn concentration in tissues, Zn-dependent enzymes activity are widely used as biomarkers of Zn status. The results of the present study showed no diet-related differences in the activity of alkaline phosphatase (AP), already considered an inadequate indicator of Zn supply in humans [45], and in dogs [46]. Similarly, plasma SOD activity, a Cu and Zn dependent enzyme, was not different in dogs fed Zn sulfate and Zn proteinate. This enzyme might be a more adequate biomarker of Zn supplementation in senior, than in young healthy dogs, since aging involves increased oxidative stress and accelerated cellular senescence, stimulating antioxidant enzymes such as catalase, CAT, glutathione peroxidase and SOD [47]. Conversely, the plasmatic concentration of alanine aminotransferase (ALT) was significantly higher in dogs fed Zn proteinate compared to Zn sulfate. The clinical importance of increased alanine and aspartate aminotransferase activities, as well as the role of Zn in the regulation of ALT in the presence of liver disease, are well known [48], whereas the significance of decreased activities is poorly understood. Nevertheless, the values herein obtained fit the interval of normality of healthy dogs [49].

Zinc can have an anti-inflammatory role by lowering the secretion of CRP, which is an acute-phase protein secreted in response to an increase in pro-inflammatory cytokines, namely of interleukin 6 [50]. Even though Zn supplementation is more efficient decreasing the CRP of ill patients, basal levels of CRP can also benefit from Zn supplementation [51]. A study of Jarosz et al. reported that chelate of Zn-glycine reduced the basal level of CRP in comparison to Zn sulfate in chickens from 1 to 42 days of age [52]. However, in the results reported here, the basal levels of CRP in dogs were not affected by Zn supplementation. 

T-cells express CD4^+^ or CD8^+^ co-receptors on their surface, defining their function. Upon activation, CD8^+^ T-cells can differentiate into cytotoxic T-cells, which recognize and eliminate virally infected and tumor cells, while CD4^+^. T-cells have helper function, meaning that they promote the function of other immune cells [53]. There is growing evidence that Zn mediates the regulation of the immune system by facilitating the transduction of signalling cascades, the physiological mechanisms being discussed elsewhere [54]. In the present study, dogs fed Zn proteinate had a significantly higher percentage of circulating CD4^+^ T-cells suggesting an improved T-cell differentiation. The positive effect of Zn on thymic function has been previously demonstrated and might provide a possible explanation for this observation [55]. In particular, the improved generation of CD4^+^ T cells has been observed in human patients undergoing oral Zn supplementation [56], in accordance with this study. The Zn source has been suggested to differently impact the number and intensity of flatulence episodes due to different production and release of H_2_S in the large intestine. Giffard et al. reported a reduction of 58% in H_2_S production with oral administration of Zn acetate, suggesting that the free Zn cations not absorbed in the small intestine bind sulfhydryl compounds such as H_2_S and methanethiol to form insoluble salts [57]. From these results, it was anticipated that inorganic Zn would reduce the number and intensity of flatulence episodes, but the values among diets were similar. This result might be, at least partially, explained by the greater bioavailability of zinc sulfate, used as the inorganic source, compared to zinc acetate used in the earlier study [58]. Nevertheless, the role of Zn in the large intestine fermentation process of dogs needs to be further investigated.

### 4.3. Exogenous Enzymes

Enzyme supplementation has long been used in poultry and pig diets to improve the nutritional value and decrease the anti-nutritional effects of NSPs, but information in dogs is much scarcer. Since enzymes degrade dietary NSPs, thus reducing their anti-nutritional effects, their use allows the decrease in dietary cost through increasing the inclusion of NSP-containing ingredients. The commercial solid-state fermentation product used in the present study comprises a multi-enzymatic complex, including phytase, protease, xylanase, ß-glucanase, cellulase, amylase and pectinase [59]. Thus, it is expected that its residual enzymatic activity could enhance the digestibility of macronutrients and minerals by degrading PA and NSP. However, the results presented here showed that CTTAD of macronutrients and energy and flatulence of dogs remained unaffected by the addition of exogenous enzymes. Conversely, a previous study with turbot juveniles observed an increase in DM digestibility, and posterior intestinal activity of lipase and protease with the addition of 400 mg/kg of the same commercial multi-enzymatic complex (Synergen^®^) used in the present study [8]. Similarly, the addition of 200 mg/kg of Synergen^®^ was related to a higher feed ratio conversion in broilers [9]. Although studies in other species have shown positive effects, the results reported here are in accordance with digestibility trials performed in adult/senior Beagle dogs. Sá et al. reported that the addition of an enzyme blend composed by 4.5 U β-glucanase/kg, 16 U xylanase/kg, 1.5 U cellulase/kg, 198 U glucoamylase/kg, 1.9 U phytase/kg, and 9000 U α-amylase/kg did not improve the digestibility of diets without and with wheat bran (25%) [60]. In line with this, Pacheco et al. reported no effects of exogenous enzymes (12/24 U amylase/kg, 16/32 U cellulase/kg, 40/80 U xylanase/kg, 80/160 U β-glucanase/kg, 120/240 U phytase/kg, 280/560 U protease/kg, and 1600/3200 U pectinase/kg) on CTTAD, fecal scores and urinary pH of dogs fed diets with 20% and 40% of full-fat rice bran [16]. The disparity in the substrate for degradation (ingredient composition of the diets) and on the level of enzymes added in each experiment preclude a direct comparison of the studies published. In the present study, the multi-enzymatic complex was added according to the recommendations for other animal species and agreeing with another study [9], in which positive effects were seen, as no recommendations are set for dogs.

In the present study, the addition of exogenous enzymes also retained the Zn bioavailability. Conversely, earlier studies reported improved Zn availability through the use of microbial phytase in broilers [61] and in pigs [62]. The results have been more evident with pigs than in broilers [63] and less consistent in dogs, which might be related to the different physiology of the gastrointestinal compartments that limits the action of the exogenous enzymes. Indeed, the success of exogenous enzymes depends upon the pH of the gastrointestinal compartment, retention times and protease activity of the host, namely pepsin, trypsin, and chymotrypsin [64,65]. Sagawa et al. reported that gastric pH of fasted dogs was 2.03, while dogs fed 10 and 200 g of dry food had on average a pH of 1.08 and 1.26, and a gastric emptying time of 562 and 1212 min, respectively [66]. Mahar et al. corroborate the inexistence of any elicit change in gastric pH 60 min after a meal (300 g of dry dog food) as pH was between 1.4 and 2.5 with occasional peaks ranging from 3.5 to 4.5 [67], whereas, another study reported that mean intestinal pH of fasted dogs was 7.3 ± 0.09 [68]. Nielsen et al. stressed the differences of activity of microbial phytases in a simulated gastric environment, highlighting the double optimum pH (3 and 6) and the decrease in activity rate of *A. niger* phytase in the presence of pepsin [69]. An in vitro study showed that the activity of β-glucanase, xylanase, amylase, and protease was optimal at pH 3–5, 5.3–6.5, 4.8 and 2, respectively [70]. Along with pH, the retention time is important for enzyme activity. According to Cuyper et al., who tested diets for dogs consisting exclusively of chunked day-old chicks, the mean total transit total time was 1692 min, of which 48.8% of the time the digest was on the stomach, 8.7% on the small intestine and 42.5% on the colon [71]. Similarly, Mahar et al. reported a gastric empty time of 971 min with 300 g of dry dog food [67]. However, ingredient composition affects retention times as reported by Pedreira et al. [72] that found increased gastric empty time after the inclusion of 10% fiber (sugarcane). It seems that the acidic pH of dogs’ stomach and the retention time might be adequate for proteases and probably reasonably adequate for phytases, being, however, detrimental for carbohydrases. In contrast, broilers have pH and retention times of 5.5 and 10–15 min in the crop, 2.5–3.5 and 30–90 min in the gizzard, 5–7 and 25–40 min in the duodenum/jejunum [73], which appears to be beneficial in terms of acidity, yet challenging due to the lower length of contact between enzymes and digesta in the various compartments. Apart from the differences in the digestion of monogastric animals, one has to consider that the success of exogenous enzymes may also vary within dogs due to the differences in the gastrointestinal physiology of breeds [74,75]. Another hypothesis that may explain the lack of effects of phytase in Zn bioavailability is the natural dissociation that acidic stomach pH of dogs allows even in the absence of phytases. If that is so, it is likely that animals with a higher stomach pH, such as pigs, could have a more clear effect of phytase activity on phytate hydrolysis [63].

## 5. Conclusions

Given the essential functions of Zn in the animal organism, the lack of information on the Zn bioavailability of the ingredients and the effect that different dietary constituents can have on the bioavailability of Zn, it is common practice to supplement diets with a Zn level above requirements to prevent symptoms of deficiency. This study evaluated, for the first time, the effect of a supplemental source of Zn added at practical levels (139 to 159 mg/kg DM), and the addition of exogenous enzymes to diets with high phytate levels on the Zn status of healthy adult dogs in maintenance. The decrease in P digestibility with inorganic Zn highlights the stability of Zn chelates in the gastrointestinal compartment, which may contribute to higher Zn bioavailability. The most commonly used Zn biomarkers (plasmatic, urinary and hair concentrations of Zn, coat quality and hair growth) remained unaffected by Zn source, but the CD4^+^T lymphocyte subset was positively affected by organic Zn supplementation, suggesting an improvement on immune function. These results highlight the need for more sensitive biomarkers, capable of detecting subclinical improvements, which although discrete can have a great impact on the dog’s health and performance. Additionally, the effects of Zn sources should be evaluated in circumstances of high Zn requirements. The addition of exogenous enzymes kept unaffected the digestibility of nutrients and phytic acid, that could be due to the low pH of the stomach of dogs that may simultaneously impair the enzymatic activity and stimulate the dissociation of phytates. More research is needed to understand the benefits of exogenous enzymes and to refine existing products to fit the unique physiology and nutritional needs of dogs.

## Figures and Tables

**Table 1:** Ingredient composition of the studied diets and zinc and phytic acid (PA) contents of individual ingredients of the four experimental diets.

Ingredient	g/kg, as is	Zn, mg/kg Dry Matter (DM)	PA, mg/kg DM
Wheat bran	200	87.3	34.8
Wheat	164	27.5	10.6
Poultry by-product meal	125	115	-
Maize	104	13.8	8.25
Soybean protein concentrate	100	52.3	21.5
Animal fat	60	-	-
Broken rice	50	17.3	1.92
Pea starch	50	24.3	10.9
Hydrolyzed salmon protein	50	-	-
Palatability enhancer	40	-	-
NuPro^®^ Yeast	30	-	-
Alfalfa protein concentrate	25	19.4	2.47
Sugar beet pulp	25	2.4	1.10
Premix ^1^	15	-	-
*Ascophyllum nodosum*	10	32.6	1.01
Mono-ammonium phosphate	8.0	-	-
Milled salt	4.0	-	-
Preservative	4.0	-	-
Sodium hexametaphosphate	0.3	-	-
Zinc ^2^ (mg/kg)	75	-	-
Solid-state fermentation product of *Aspergillus niger* ^3^ (mg/kg)	0/200	-	-

^1^ Premix per kg of diet: vitamin A 14,950 UI; vitamin D_3_ 1560 UI; vitamin E 98.0 mg; thiamine 2 mg; riboflavin 4 mg, niacin 30 μg; cobalamin 30 μg; vitamin B_6_ 3 mg; folic acid 495 μg; biotin 150 μg; vitamin K 2 mg; pantothenic acid 20 mg; CuSO_4_·5H_2_O 8 mg; KI 2 mg; MnSO_4_·H_2_O 5 mg; Na_2_SeO_3_ 100 μg; ^2^ Diets IZ and IZ+ were supplemented with 206 mg/kg of ZnSO_4_.H_2_O while diets OZ and OZ+ with 5 g/kg of zinc proteinate (Bioplex^®^); ^3^ Diets IZ and OZ were not supplemented with a solid-state fermentation product of *Aspergillus niger* (Synergen^®^), and diets IZ+ and OZ+ were supplemented with 200 mg/kg of Synergen^®^.

**Table 2:** Antibodies for cytokine intracellular staining.

Antibody	Species	Stain	Dilution	Brand
CD3	Mouse anti-dog	FITC	1:10	Bio-Rad Laboratories (Irvine, CA, USA)
CD4	Anti-canine	PE/Cy7	1:100	eBioscience
CD8	Rat anti-dog	Alexa700	1:10	Bio-Rad Laboratories

**Table 3:** Chemical composition of the experimental diets.

Units/kg DM	Diets			
	IZ	IZ+	OZ	OZ+
DM, g	922	930	910	911
Ash, g	71.9	69.6	70.9	75.3
CP, g	274	280	288	291
EE, g	120	118	115	111
NDF, g	191	212	217	207
ADF, g	39.5	44.4	49.2	47.4
ADL, g	35.1	36.0	38.5	35.6
Starch, g	310	310	287	284
GE, MJ	19.9	20.3	20.0	20.1
Ca, g	15.6	15.2	16.5	17.1
Total P, g	11.5	10.4	12.1	10.7
Ca:P	1.35	1.46	1.37	1.60
Phytic acid, g	10.6	9.70	11.0	9.70
Mg, g	1.34	1.34	1.41	1.41
Na, g	6.03	5.31	6.34	5.96
K, g	7.43	7.27	7.61	7.62
Fe, mg	312	300	311	303
Zn, mg	159	139	140	150
Mn, mg	57.8	57.9	58.5	58.7
Cu, mg	21.4	18.8	18.8	19.5
Se, µg	537	513	513	565

**Table 4:** Body condition score (BCS) and daily intake of macronutrients, minerals, metabolizable energy (ME), and phytic acid (PA) of dogs fed the experimental diets. ^a^^–c^ Values in the same row that share a common superscript are not statistically different (*p* > 0.05).

Parameter	Diets					*p*		
	IZ	IZ+	OZ	OZ+	SEM	Zn	Enzyme	Zn × Enzyme
BCS	4.5	4.6	4.5	4.4	0.10	1.000	0.624	0.624
Intake, units/kg BW^0.75^								
DM, g	42.0 ^a^	40.3 ^b^	39.5 ^b^	40.4 ^ab^	0.60	0.063	0.490	0.032
OM, g	39.0	37.5	36.7	37.4	0.56	0.044	0.442	0.054
CP, g	11.3	11.1	11.3	11.7	0.17	0.105	0.532	0.056
Starch, g	12.8	12.3	11.3	11.4	0.18	<0.001	0.377	0.061
EE, g	4.96	4.69	4.51	4.47	0.071	<0.001	0.031	0.107
NDF, g	7.98 ^a^	8.59 ^b^	8.73 ^b^	8.54 ^b^	0.098	0.001	0.039	<0.001
Ca, g	0.646	0.635	0.647	0.689	0.0190	0.158	0.414	0.174
P, g	0.476	0.413	0.474	0.431	0.0068	0.237	<0.001	0.156
Fe, mg	12.9 ^a^	11.9 ^b^	12.2 ^b^	12.2 ^ab^	0.19	0.260	0.013	0.010
Cu, mg	0.886 ^a^	0.746 ^bc^	0.734 ^b^	0.786 ^c^	0.0122	<0.001	0.001	<0.001
Mn, mg	2.39 ^a^	2.30 ^b^	2.29 ^b^	2.37 ^ab^	0.035	0.638	0.759	0.024
Zn, mg	7.15 ^a^	5.95 ^b^	6.10 ^b^	6.64 ^c^	0.082	0.086	0.003	<0.001
ME, kJ	824	806	784	810	12.0	0.146	0.745	0.079
PA, g	0.439	0.385	0.431	0.391	0.0063	0.884	<0.001	0.307

**Table 5:** Digestibility of macronutrients, P and gross energy (GE) expressed as g/kg, fecal score and production of dogs fed the experimental diets. ^a,b^ Values in the same row that share a common superscript are not statistically different (*p* > 0.05).

Parameter	Diets					*p*		
	IZ	IZ+	OZ	OZ+	SEM	Zn	Enzyme	Zn × Enzyme
DM	736	742	729	728	5.6	0.078	0.646	0.493
OM	765	769	758	758	5.0	0.076	0.67	0.676
CP	711	737	725	701	19.1	0.553	0.943	0.197
Starch	994	993	993	994	0.9	0.901	0.645	0.345
EE	941	939	937	938	1.6	0.147	0.596	0.419
NDF	444 ^a^	496 ^b^	471 ^ab^	456 ^ab^	12.8	0.639	0.162	0.013
GE	767	771	763	762	5.9	0.287	0.798	0.622
P	273	276	332	372	27.8	0.009	0.447	0.493
Fecal score	3.2	3.2	3.3	3.3	0.10	0.121	0.654	0.852
Fecal production, g DM	325	321	324	330	7.1	0.586	0.844	0.493

**Table 6:** Concentration of Zn in hair (mg/kg) and urine (μg/g CT), plasmatic concentration of trace elements (unit per g of creatinine, CT), activity of superoxide dismutase (SOD), alanine aminotransferase (ALT), C-reactive protein (CRP) and % of CD4 and CD8 cells of dogs fed the experimental diets.

Parameter	Diets					*p*		
	IZ	IZ+	OZ	OZ+	SEM	Zn	Enzyme	Zn × Enzyme
Hair [Zn], mg/kg	156	164	161	166	3.9	0.416	0.075	0.678
Urinary [Zn], μg/g CT	198	184	201	228	14.2	0.110	0.646	0.165
Plasma [Zn], mg/g CT	100	91.9	89.8	93.6	6.13	0.473	0.703	0.318
Plasma [Cu], mg/g CT	76.3	75.6	70.9	77.0	4.52	0.660	0.560	0.458
Plasma [Mn], μg/g CT	572	562	519	573	30.1	0.496	0.467	0.295
Plasma [Fe], mg/g CT	589	540	534	524	38.8	0.376	0.454	0.625
SOD, U/g TP	880	862	955	975	81.9	0.261	0.991	0.821
ALT, U/L	31.5	31.4	33.9	34.3	1.20	0.035	0.914	0.887
CRP, mg/L	1.62	1.94	1.92	1.84	0.272	0.723	0.656	0.476
CD4, %	62.8	64.2	65.3	65.3	0.85	0.041	0.379	0.401
CD8, %	19.0	19.0	18.4	19.1	0.57	0.655	0.609	0.526
CD4:CD8 ratio	3.45	3.52	3.74	3.61	0.115	0.109	0.810	0.395

**Table 7:** Visual and sensorial coat evaluation and hair growth assessment (trichogram) of dogs fed the experimental diets.

Parameter	Diets					*p*		
	IZ	IZ+	OZ	OZ+	SEM	Zn	Enzyme	Zn × Enzyme
Coat evaluation								
Gloss	4.1	4.1	4.3	4.1	0.08	0.061	0.267	0.245
Softness	3.8	3.9	3.8	3.8	0.11	0.918	0.720	0.817
Greasiness	3.9	4.0	4.0	4.1	0.07	0.369	0.313	0.851
Scale	3.9	3.9	3.8	3.9	0.10	0.709	0.327	0.632
Trichogram								
Anagen, %	9.12	13.7	14.4	12.3	2.07	0.352	0.498	0.119
Catagen, %	1.72	3.08	3.19	2.52	0.775	0.555	0.666	0.200
Telogen, %	89.2	83.2	82.4	85.2	2.26	0.293	0.491	0.065

**Table 8:** The number of episodes, average and maximum intensity (H_2_S detected) of flatus of dogs fed the experimental diets.

Parameter	Diets					*p*		
	IZ	IZ+	OZ	OZ+	SEM	Zn	Enzyme	Zn × Enzyme
Number of episodes	10.3	6.0	6.0	3.3	3.33	0.320	0.320	0.827
Average intensity, ppm	17.0	11.0	13.2	12.9	2.20	0.572	0.242	0.220
Max intensity, ppm	33.8	20.4	23.6	21.8	6.57	0.378	0.366	0.406

## Supplementary Materials

The following are available online at https://www.mdpi.com/2076-2615/10/3/400/s1, Figure S1: Graphical representation of the gating path followed to obtain CD4 and CD8 subsets, Table S1: Analysis of certified reference material (BCR^®^-679, ERM^®^-DB001 and oat flour powder), Table S2: Analysis of certified reference material (Seronorm™ Urine and Seronorm™ Serum).
