# Supplementary material for: Effect of Zinc Source and Exogenous Enzymes Supplementation on Zinc Status in Dogs Fed High Phytate Diets
Source: Animals (Basel). 2020 Feb 29;10(3):400. doi: 10.3390/ani10030400 (PMC7142709; doi:10.3390/ani10030400)
Supplement: Supplementary file 1 [file animals-10-00400-s001.pdf]

Table S1. Certified and measured values of certified reference materials BCR®-679, ERM®-DB001 and oat flour powder

Element Units/kg	BCR®-679		ERM®-DB001		Oat Flour Powder[c]	
	Certified Value	Measured Value	Certified Value	Measured Value	Certified Value	Measured Value
Ca, g	7.8 ± 0.7 [I]	7.53	---[b]	---[b]	---[b]	---[b]
P, g	3.3 ± 0.2 [I]	---[b]	---[b]	---[b]	4.35	4.40 ± 0.1
Mg, g	1.4 ± 0.1 [I]	1.34	---[b]	---[b]	---[b]	---[b]
K, g	---[b]	---[b]	---[b]	---[b]	---[b]	---[b]
Fe, mg	55.0 ± 2.5	---[b]	---[b]	---[b]	---[b]	---[b]
Zn, mg	79.7 ± 2.7	76.7 ± 2.44	209 ± 12	201 ± 5.7	---[b]	---[b]
Mn, mg	13.3 ± 0.5	14.0 ± 0.34	---[b]	---[b]	---[b]	---[b]
Cu, mg	2.89 ± 0.12	2.92 ± 0.02	33 ± 4	---[b]	---[b]	---[b]

[b] Not available or not quantified; [I] – Indicative values; [c] – supllied with Phytic Acid Assay Kit (Megazyme),

Table S2. Analytical uncertainty with 95 % confidence interval provided and measured values (ppb) of certified reference materials Seronorm™ Urine and Seronorm™ Serum

Element ppb	Level	Seronorm™ Urine		Seronorm™ Serum	
		Analytical Uncertainty	Measured Value	Analytical Uncertainty	Measured Value
Fe	1	---[b]	---[b]	1170 - 1770	1561
	2	---[b]	---[b]	1720 - 2580	2371
Zn	1	277 - 417	217	952 - 1242	805
	2	1023 - 1538	1257	1404 - 1831	1543
Mn	1	1.10 – 1.66	1.52	7.9 – 11.9	8.07
	2	7.4 – 11.2	10.3	11.6 – 17.4	13.3
Cu	1	16 - 24	24.9	999 - 1281	1110
	2	44.9 - 67.6	57.6	1700 - 2000	1978

[b] Not available or not quantified

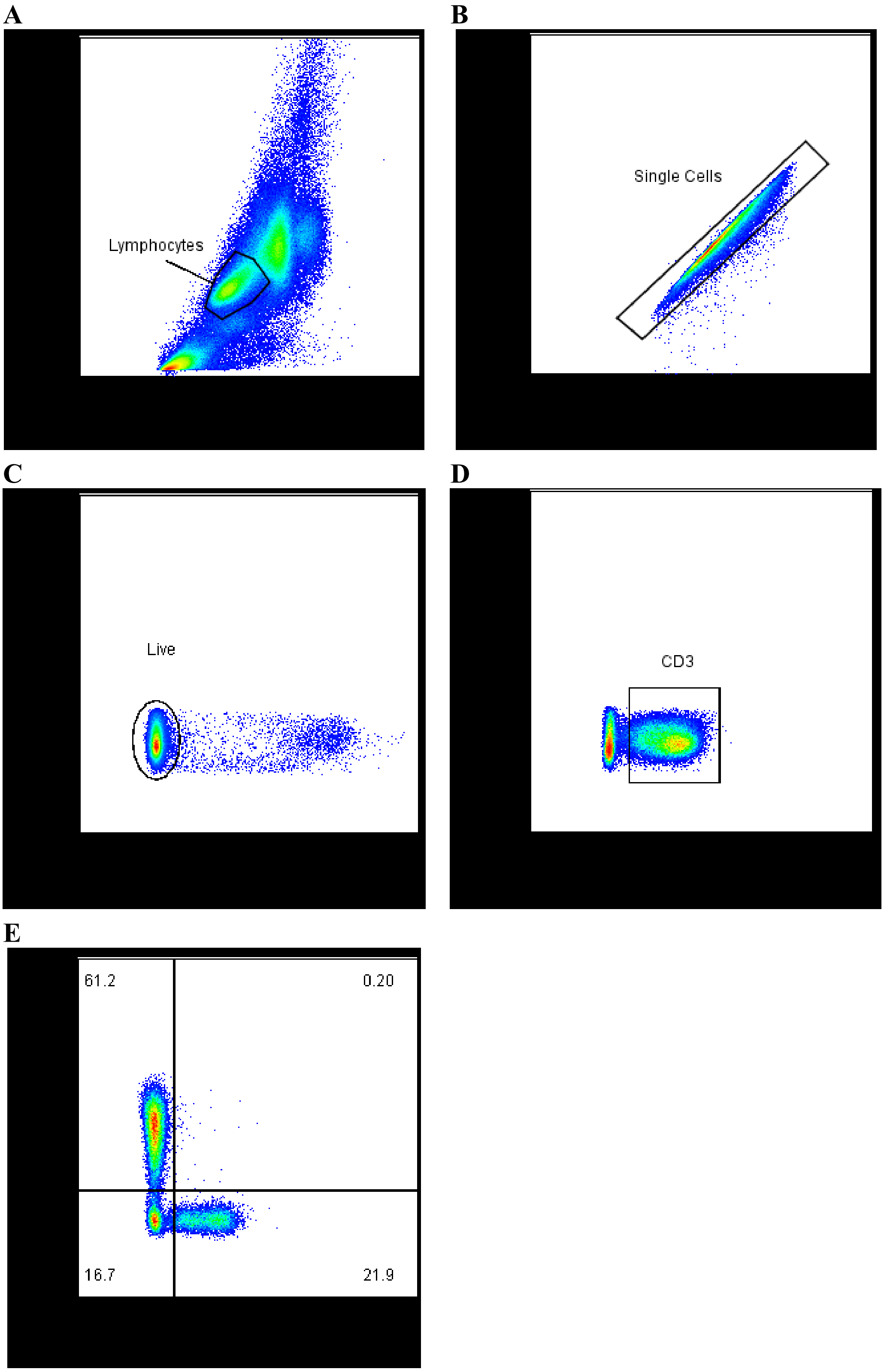

Figure S1. Representative example of the gating strategy followed to obtain CD4+ and CD8+ T cell subsets. Lymphocytes (A) were selected based on FSC and SSC parameters, as cells presenting small size and low granularity; single cells (B) were defined by plotting FSC-A *vs* FSC-H ; and live cells (C) were selected among cells that did not incorporate propidium iodide; T cells were defined as CD3+ (D); Single CD4+ (up-left quadrant) and CD8+ (low-right quadrant) T cells (E) were set according to their expression of CD4 and CD8, respectively. Values inside the dot plot correspond to the percentage of cells in each quadrant. Gates were defined based on FMO control stainings.
